# Supplementary material for: Collective movement of schooling fish reduces the costs of locomotion in turbulent conditions
Source: PLoS Biol. 2024 Jun 6;22(6):e3002501. doi: 10.1371/journal.pbio.3002501 (PMC11156351; doi:10.1371/journal.pbio.3002501)
Supplement: S1 Text — Fig A. Photograph of the passive turbulence grid used to generate the turbulent flows for the fish swimming section inside the swim-tunnel respirometer. Each opening is 1.5 × 1.5 cm. The plate has a height and width of 7.5 × 8.6 cm. The turbulence grid is inserted in front of the fish swimming section. The pass of the laminar-like flow through the grid generates higher velocity jets through the openings. The jets of flow then mix and create a turbulent environment for the entire swimming section (see S2 Fig for the characterizations of the turbulence in S1 Text). Fig B. Characteristics of the turbulence generated by a passive grid for different mean velocities. (A) Transverse (DNN) and longitudinal (DLL) structure functions for 2, 4, 6, 8 BLs−1. In the inertial subrange, the structure functions scale as r2/3 which agrees with Kolmogorov’s local isotropy hypothesis. (B) Energy dissipation rate is computed from DNN and DLL using the relationship shown. The energy dissipation rate was approximated as the average maximum value between the 2 curves. (C) One-dimensional energy spectrum for each mean flow tested. The x-axis denotes the wavenumber (K) normalized by the integral length scale. The y-axis denotes the amount of energy contained in eddies of a certain wavenumber. The energy is normalized by the integral length scale times the square of the total fluctuation velocity. The dashed line demonstrates that the energy scales as the wavenumber to the power minus five-thirds, agreeing with Kolmogorov’s hypothesis. The underlying data of this figure are in doi.org/10.7910/DVN/CVNLZE. Fig C. The mathematical relationship between average water velocity and revolutions per minute in the swim-tunnel respirometer. The average water velocity (denoted as “y”) is measured at the swimming section of the respirometer by particle image velocimetry (PIV). The revolutions per minute (RPM, denoted as “x”) is the rotation speed of the motor on the swim-tunnel respirometer. The average w [file pbio.3002501.s001.docx]

Supplementary Materials 1 for

Collective movement of schooling fish reduces the costs of locomotion in turbulent conditions

Yangfan Zhang^1^*, Hungtang Ko^2^, Michael Calicchia^3^, Rui Ni^3^, George V. Lauder^1^

^1^Department of Organismic and Evolutionary Biology, Harvard University, 26 Oxford St, Cambridge, Massachusetts, 02138, USA

^2^Department of Mechanical and Aerospace Engineering, Princeton University, Olden St., Princeton, New Jersey, 08540, USA

^3^Department of Mechanical Engineering, Johns Hopkins University, Baltimore, MD-21218, USA

*Corresponding author. Email: yangfan_zhang@fas.harvard.edu

**This PDF file includes:**

Introductory text p. 2

Figs A-I in S1 Text pp. 3 – 13

Computing fluctuation velocity and turbulence intensity pp. 5

Determining eddy size distribution in turbulence flow pp. 6

Tables A-B in S1 Text pp. 14 – 16

Introductory Text to Supplemental Materials

Below we provide supplementary figures and supplementary tables. These materials are a supplement to the main text and explain the methods in detail, along with supplemental figures that document how we create and quantify the fluid dynamic features of turbulent flows. We also describe the measurements and modelling method to quantify the aerobic and non-aerobic contributions to the total energy expenditure of swimming in turbulent and laminar flows both by individuals and by the replicate schools of giant danio. Some of the supplemental materials are adopted from a previous study since the two studies used the same experimental system.


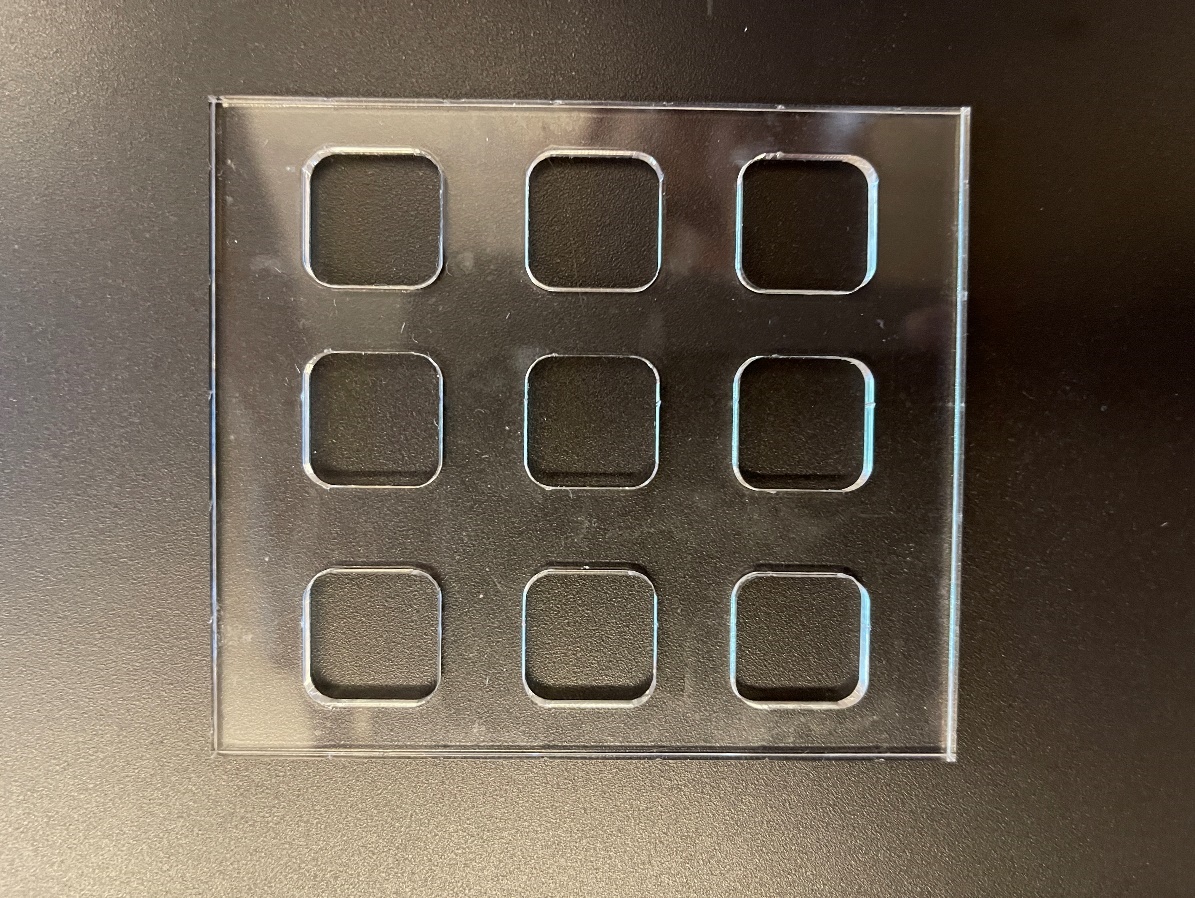


**Fig A. Photograph of the passive turbulence grid used to generate the turbulent flows for the fish swimming section inside the swim-tunnel respirometer.** Each opening is 1.5 × 1.5 cm. The plate has a height and width of 7.5 × 8.6 cm. The turbulence grid is inserted in front of the fish swimming section. The pass of the laminar-like flow through the grid generates higher velocity jets through the openings. The jets of flow then mix and create a turbulent environment for the entire swimming section (*see* Fig B in S1 Text for the characterizations of the turbulence).


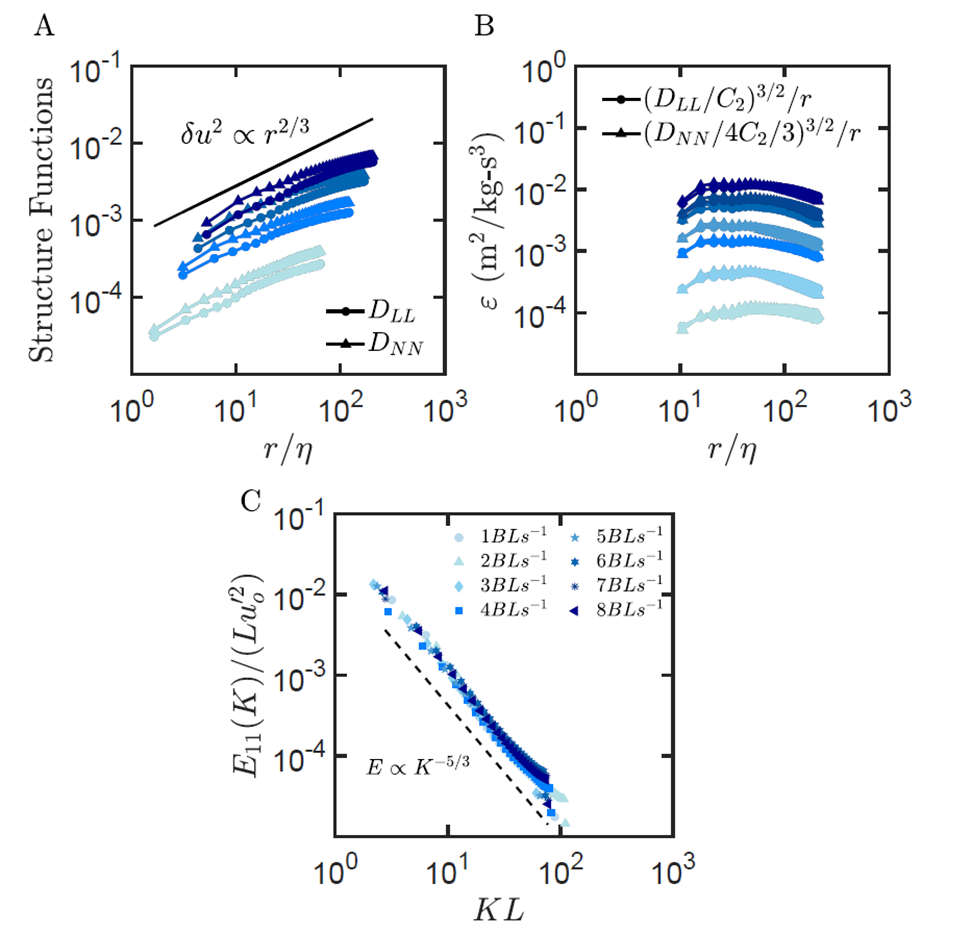


**Fig B.** **Characteristics of the turbulence generated by a passive grid for different mean velocities.** (A) Transverse $(D_{NN})$ and longitudinal $(D_{LL})$ structure functions for $2, 4, 6, 8 BLs^{-1}$. In the inertial subrange, the structure functions scale as $r^{2/3}$ which agrees with Kolmogorov’s local isotropy hypothesis. (B) Energy dissipation rate is computed from $D_{NN}$ and $D_{LL}$ using the relationship shown. The energy dissipation rate was approximated as the average maximum value between the two curves. (C) One-dimensional energy spectrum for each mean flow tested. The x-axis denotes the wavenumber $(K)$ normalized by the integral length scale. The y-axis denotes the amount of energy contained in eddies of a certain wavenumber. The energy is normalized by the integral length scale times the square of the total fluctuation velocity. The dashed line demonstrates that the energy scales as the wavenumber to the power minus five- thirds, agreeing with Kolmogorov’s hypothesis.

**Computing fluctuation velocity and turbulence intensity**

To quantify the level of turbulence present at each swimming speed, the fluctuation velocity and turbulence intensity were computed. $u^{'}$is the fluctuation velocity in the direction parallel to the swimming direction. It is computed by taking the standard deviation of the component of the entire velocity field parallel to the swimming direction over space and time. The equation is shown below. Here, *N* is equal to the number of velocity vectors at a given time step multiplied by the number of time steps, and $\left\langle u \right\rangle$ is the mean velocity in the swimming direction.

$$u^{'}=\sqrt{\frac{1}{N-1}\sum_{i=1}^{N} \left( u_{i}-\left\langle u \right\rangle\right)^{2}}$$

$v^{'}$ is the fluctuation velocity perpendicular to the swimming direction. It is computed by taking the standard deviation of the component of the entire velocity field perpendicular to the swimming direction over space and time.

$$v^{'}=\sqrt{\frac{1}{N-1}\sum_{i=1}^{N} \left( v_{i}-\left\langle v \right\rangle\right)^{2}}$$

$u_{o}^{'}$ is the total fluctuation velocity, which scales as the square root of two-thirds of the turbulent kinetic energy ($k$). Since information on the velocity field only exists on a 2D plane, it is assumed that the total fluctuation velocity scales as the square root of the turbulent kinetic energy.

$$u_{o}^{'}=\sqrt{k}=\sqrt{\frac{1}{2}{[u^{'}}^{2}+{v^{'}}^{2}]}$$

$I_{x}$ and $I_{y}$ represent the turbulence intensity in the direction parallel and perpendicular to the swimming direction, respectively, and $I$ is the total turbulence intensity. They are computed as shown below.

$$I_{x}=\frac{u^{'}}{\left\langle u \right\rangle}$$

$$I_{y}=\frac{v^{'}}{\left\langle u \right\rangle}$$

$$I=\frac{u^{'}+v^{'}}{2\left\langle u \right\rangle}$$

**Determining eddy size distribution in turbulent flow**

To determine the scale separation, first the longitudinal $(D_{LL})$ and transverse $(D_{NN})$ structure functions were computed, where $r$ denotes the distance between two velocity vectors, $u_{l}$ denotes the velocity projected along the direction of $r$, and $u_{n}$denotes the velocity perpendicular to the direction of $r$. Here, $\left\langle\cdot\right\rangle$ denotes an average over all velocity pairs that have the same separation distance $r$

$$D_{LL}\left( r \right)=\left\langle\left[ u_{l}\left( x \right)-u_{l}\left( x+r \right) \right]^{2} \right\rangle$$

$$D_{NN}\left( r \right)=\left\langle\left[ u_{n}\left( x \right)-u_{n}\left( x+r \right) \right]^{2} \right\rangle$$

Using Kolmogorov’s local isotropy hypothesis, the structure functions can be related to the energy dissipation rate ($\varepsilon)$through the following relationships, where $C_{2}$ is a universal constant and is assumed to be 2.

$$D_{LL}\left( r \right)=C_{2}\left( \varepsilon r \right)^{\frac{2}{3}}$$

$$D_{NN}\left( r \right)=\frac{4}{3}C_{2}\left( \varepsilon r \right)^{2/3}$$

These equations can be rearranged to solve for energy dissipation rate. In the inertial subrange, the energy dissipation rate curves plateau to a maximum value. The energy dissipation rate was then approximated as the average maximum value between the two curves. After obtaining an approximation of the energy dissipation rate from the 2D structure functions, the integral and Kolmogorov length scales can be approximated as follows, where $u_{o}^{'}$ is the total fluctuation velocity and $\nu$ is the kinematic viscosity of water.

$${\eta=\left( \frac{\nu^{3}}{\varepsilon} \right)}^{\frac{1}{4}}$$

$$L= \frac{\left( u_{o}^{'} \right)^{3}}{\varepsilon}$$

The one-dimensional energy spectrum was computed as follows. First, the non-dimensional longitudinal correlation function $(f$) was computed as shown below, where $u_{l}^{'}$ denotes the fluctuation velocity projected along the direction of $r$

$$f\left( r \right)=\frac{\left\langle\left[ u_{l}^{'}\left( x \right)][u_{l}^{'}\left( x+r \right) \right] \right\rangle}{\left\langle{u^{'}}_{l}^{2}(x) \right\rangle}$$

Then, the energy spectrum can be obtained by taking the Fourier transform of $f\left( r \right)$ and multiplying it by the variance.

**Fig C. The mathematical relationship between average water velocity and revolutions per minute in the swim-tunnel respirometer.** The average water velocity (denoted as ‘y’) is measured at the swimming section of the respirometer by particle image velocimetry (PIV). The revolutions per minute (RPM, denoted as ‘x’) is the rotation speed of the motor on the swim-tunnel respirometer. The average water velocity in the swimming section is quantified in both laminar (green) and turbulent (orange) flow conditions. The linear regression equations are stated in the figure legends.

**Fig D. A schematic illustration of the incremental steps in swimming speed used for the critical swimming speed test (*U*_crit_) protocol.** Speed is presented as the relative swimming speed of fish normalized to body lengths per second (BL s^-1^). Each speed increment has a 10-min duration. Each vertical increment marks the increase in speed for each step. The mean velocity at fatigue is indicated by the * symbol, marking the total duration of the test. The methodological details are adapted from [62] Zhang, Y., & Lauder, G. V. (2024), eLife, 12, doi:10.7554/eLife.90352.2. as both studies used the same experimental system and similar experimental protocols.

Fig E. Stability of whole-animal oxygen uptake (*Ṁ*O_2_) profile when fish swim steadily at low water velocity. The quality of *Ṁ*O_2_ measurement over the range of relative swimming speeds (body length per sec, BL s^-1^) where fish show the lowest *Ṁ*O_2_ is critical to assure the accuracy of the *J*-shaped metabolism-speed curve. Hence, we conducted an additional quality assurance test to inspect the stability of *Ṁ*O_2_ at 1.25 (A), 1.5 (B), 1.75 (C) & 2.0 (D) BL s^-1^ over a 30-min period. *Ṁ*O_2_ recorded in the first 10 mins is within the same range as the ensuing 20 mins. Thus, our 10-min testing period at each water velocity provides a reliable estimate of the aerobic cost when fish swim at a given swimming speed. This conclusion is in agreement with the same testing period used in a previous study ([64] V. Di Santo et al., 2017 PNAS 114, 13048–13053). The methodological details are adapted from [62] Zhang, Y., & Lauder, G. V. (2024), elife, 12, doi:10.7554/eLife.90352.2 as both studies used the same experimental system and similar experimental protocols.

Fig F. An analysis of the impact of varying the sampling window duration on estimates of individual rates of oxygen uptake (*Ṁ*O_2_). The signal-to-noise ratio analyses used six independent background respirometer *Ṁ*O_2_ data sets in a 20-min duration. We calculated (A) standard deviations (S.D.) and (B) coefficient of variation (C.V.) for each complete data set as a function of sampling window duration (0.8 to 5.0 min). The S.D. and C.V. values were compared across different sampling window durations using one-way ANOVA with Tukey *post-hoc* tests (𝛼 < 0.05). Values are presented as mean ± s.e.m.. This analysis suggests that 1.67-min as a minimum conservative sampling duration (the red vertical dashed line) using the criterion of S.D. and C.V. being stable after high variation at shorter duration windows. This suggests that the 8-min sampling duration used in this study is more than sufficient to resolve the measurement of steady-state *Ṁ*O_2_ for sustained swimming. The methodological details are adapted from [62] Zhang, Y., & Lauder, G. V. (2024), elife, 12, doi:10.7554/eLife.90352.2 as both studies used the same experimental system and similar experimental protocols.

**Fig G. Illustration of the theoretical relationship between the cost of transport and total power as a function of nominal speed based on the estimated power required to swim as drag forces increase at increasing swimming speeds.** The equation for the energy cost of transport is y = 0.9996x^-1^ + x^2^ (power series model: R^2^ = 1, AIC = -127.6). The equation of total power is y = 1.001 – 0.000643x + 8.564e^-005^x^2^ + 1x^3^ (polynomial model: R^2^ = 1, AIC = -138.7). Notably, the same models provided best-fit equations to describe the measurement of the cost of transport and total energy expenditure (aerobic and glycolytic metabolism) on live fish. This suggests that total energy expenditure during fish locomotion at increasing speed may be largely due to the requirement to overcome fluid dynamic drag. The theoretical numbers (nominal) of power and energy cost of transport are adopted from D. Robinson, Ed., Animal Performance (The Open University, Milton Keynes, 1997). Speed is given in body lengths per second (BL s^-1^) while the power and energy axes are unitless relative numbers. The methodological details are adapted from [62] Zhang, Y., & Lauder, G. V. (2024), eLife, 12, doi:10.7554/eLife.90352.2 as both studies used the same experimental system and similar experimental protocols.

**Fig H. Photographs of the ‘spike’ calibration device used to map the three-dimensional space of the fish swimming section inside the swim-tunnel respirometer.** This direct linear transformation calibration system provided accurate 3D coordinates within the swimming arena for both individual danio and schools of up to 8 danio. The tip of each spike provides a marker for spatial coordinates. Each tip has a known three-dimensional coordinate based on the known distance on the x-(length), y-(width) & z-(height) axes. Lateral (**A**) and dorsal (**B**) views are provided. The ‘spike’ calibration provides 45 spatial points to sufficiently cover a volume of 1440•10^3^ mm^3^. The spatial calibration is used by DLTdv8 software (Hedrick, T. L. 2008, Bioinspiration & Biomimetics 3, 034001) to extract the three-dimensional coordinates for locations digitized on individual fish (see Fig I in S1 Text). The methodological details are adapted from [62] Zhang, Y., & Lauder, G. V. (2024), eLife, 12, doi:10.7554/eLife.90352.2 as both studies used the same experimental system and similar experimental protocols.


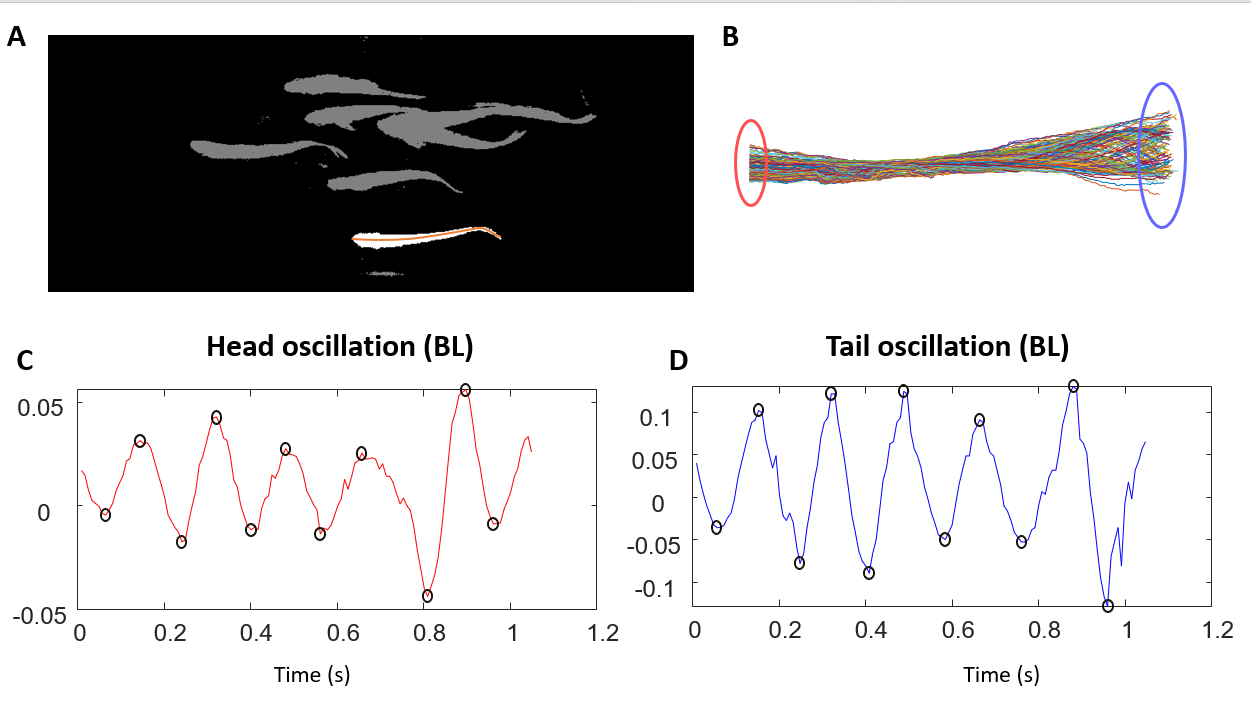


**Fig I. Extractions of individual fish kinematics in the schools.** (A) A representative video frame converted into black and white. The region and the midline representing the fish being tracked are highlighted. (B) Midlines obtained through processing consecutive video frames. Time series of (C) head and (D) tail oscillations in periodic patterns. The peaks and troughs are marked in black circles.

**Table A.** Parameters for the metabolic model for calculating the total O_2_ cost during a critical swimming speed (*U*_crit_) test in turbulent conditions. This model adds the amount of O_2_ consumed post *U*_crit_ test (excess post-exercise O_2_ consumption, EPOC, calculated by an area under the curve algorithm, AUC) on the active O_2_ uptake (*Ṁ*O_2_) over 2–7 body lengths per second (BL s^-1^) when fish use ≥ 20% maximum *Ṁ*O_2_ (*Ṁ*O_2max_). A threshold level of ~40% *Ṁ*O_2max_ is the workload that initiates glycolytic metabolism in fish schools, whereas the modelling indicated that the glycolytic metabolism is initiated at ~27% *Ṁ*O_2max_ in solitary individuals. Glycolytic metabolic cost is a major contributor to EPOC in addition to the use of high-energy phosphates. The model uses a percentage (%) modifier to compute the O_2_ cost in addition to the active *Ṁ*O_2_ at each swimming speed. The key criterion is that the area of non-aerobic O_2_ cost above the *Ṁ*O_2_ should be equal to EPOC, as shown in the section of the table as EPOC validation. The added area of non-aerobic O_2_ cost is calculated by the delta of AUC for the measured *Ṁ*O_2_ and the AUC for the *Ṁ*O_2_ model. As a result, the calculated total energy expenditure is modelled by the same equation for the theoretical relationship between total power and swimming speed (Fig G in S1 Text). Modelling is performed on each school and each solitary individual to calculate the 95% C.I. and the group average is presented in Fig 4. The modelling for the aerobic and anaerobic metabolic contribution of *D. aequipinnatus* to swim in the laminar flows is available in [62] Zhang, Y., & Lauder, G. V. (2024), eLife, 12, doi:10.7554/eLife.90352.2.

|  | **Model parameters** | | | |  | **EPOC validation** | |  |  |  |
| --- | --- | --- | --- | --- | --- | --- | --- | --- | --- | --- |
|  | Swim speed  (BL s^-1^) | *Ṁ*O_2_ (mg O_2_h^-1^kg^-1^) | Modeled *Ṁ*O_2_ (mg O_2_h^-1^kg^-1^) | % Modifier | %  *Ṁ*O_2max_ | AUC of measure (mg O_2_ kg^-1^) | AUC of model (mg O_2_ kg^-1^) | Delta AUC (model-measure) (mg O_2_ kg^-1^) | EPOC (mg O_2_ kg^-1^) | % Difference of Delta AUC & EPOC |
|  |  |  |  |  |  |  |  |  |  |  |
| School 1 | 3 | 416 | 478 | 15 | 46% | 990 | 1241 | 251 | 251 | 0% |
|  | 4 | 530 | 663 | 25 | 59% |  |  |  |  |  |
|  | 5 | 642 | 937 | 46 | 71% |  |  |  |  |  |
|  | 6 | 807 | 1340 | 66 | 90% |  |  |  |  |  |
|  | 7 | 899 | 1862 | 107 | 100% |  |  |  |  |  |
| School 2 | 3 | 360 | 450 | 25 | 36% | 816 | 1176 | 360 | 360 | 0% |
|  | 4 | 410 | 599 | 46 | 41% |  |  |  |  |  |
|  | 5 | 606 | 1006 | 66 | 60% |  |  |  |  |  |
|  | 6 | 690 | 1470 | 113 | 69% |  |  |  |  |  |
|  | 7 | 1002 | 2404 | 140 | 100% |  |  |  |  |  |
| School 4 | 3 | 304 | 441 | 45 | 39% | 649 | 1205 | 556 | 555 | -0.1% |
|  | 4 | 350 | 687 | 96 | 45% |  |  |  |  |  |
|  | 5 | 475 | 1140 | 140 | 61% |  |  |  |  |  |
|  | 6 | 682 | 1883 | 176 | 88% |  |  |  |  |  |
|  | 7 | 779 | 2766 | 255 | 100% |  |  |  |  |  |
| School 5 | 3 | 388 | 481 | 24 | 42% | 690 | 1151 | 461 | 462 | 0.2% |
|  | 4 | 413 | 627 | 52 | 45% |  |  |  |  |  |
|  | 5 | 581 | 987 | 70 | 63% |  |  |  |  |  |
|  | 6 | 698 | 1737 | 149 | 76% |  |  |  |  |  |
|  | 7 | 922 | 2940 | 219 | 100% |  |  |  |  |  |
| Individual 1 | 2 | 316 | 947 | 200 | 29% | 815 | 4760 | 3945 | 3938 | -0.2% |
|  | 2.5 | 382 | 1872 | 390 | 35% |  |  |  |  |  |
|  | 3 | 527 | 2844 | 440 | 48% |  |  |  |  |  |
|  | 4 | 479 | 5080 | 960 | 44% |  |  |  |  |  |
|  | 5 | 656 | 8496 | 1195 | 60% |  |  |  |  |  |
|  | 6 | 1086 | 14662 | 1250 | 100% |  |  |  |  |  |
| Individual 2 | 2.5 | 236 | 661 | 180 | 21% | 1036 | 4515 | 3479 | 3479 | 0% |
|  | 3 | 368 | 1583 | 330 | 33% |  |  |  |  |  |
|  | 4 | 614 | 3315 | 440 | 56% |  |  |  |  |  |
|  | 5 | 577 | 5766 | 900 | 52% |  |  |  |  |  |
|  | 6 | 1101 | 7931 | 620 | 100% |  |  |  |  |  |
|  | 7 | 903 | 9938 | 1000 | 82% |  |  |  |  |  |
| Individual 3 | 2.5 | 304 | 559 | 84 | 30% | 896 | 3228 | 2332 | 2332 | 0% |
|  | 3 | 227 | 577 | 154 | 22% |  |  |  |  |  |
|  | 4 | 571 | 1794 | 214 | 56% |  |  |  |  |  |
|  | 5 | 829 | 3929 | 374 | 81% |  |  |  |  |  |
|  | 6 | 1019 | 5847 | 474 | 100% |  |  |  |  |  |
|  | 7 | 1476 | 9949 | 574 | 145% |  |  |  |  |  |

**Table B.** Energetic contributions from aerobic and glycolytic metabolism for swimming in turbulent conditions. The partitions of metabolic contribution (mean ± s.e.m.) are detailed for giant danio (*Devario aequipinnatus*) to swim at each testing speed that engages the glycolytic metabolism. The values are calculated from the metabolic modelling (Table A). The partitions of aerobic and anaerobic metabolic contribution for *D. aequipinnatus* to swim in the laminar flows are available in [62] Zhang, Y., & Lauder, G. V. (2024), eLife, 12, doi:10.7554/eLife.90352.2.

| Swim speed (BL s^-1^) | 2.5 | 3 | 4 | 5 | 6 | 7 |
| --- | --- | --- | --- | --- | --- | --- |
| Aerobic contribution in fish schools (%) |  | 79.1±3.7 | 66.3 ±6.0 | 57.3 ±5.6 | 45.9±5.3 | 37.4±4.6 |
| Anaerobic contribution in fish schools (%) |  | 20.9±3.7 | 33.7±6.0 | 42.7±5.6 | 54.1±5.3 | 62.6±4.6 |
| Aerobic contribution in solitary fish (%) | 39.1±7.7 | 26.8±6.3 | 22.5±4.7 | 13.3±3.9 | 12.8±2.8 | 10.3±2.3 |
| Anaerobic contribution in solitary fish (%) | 60.9±7.7 | 73.2±6.3 | 77.5±4.7 | 86.7±3.9 | 87.2±2.8 | 89.7±2.3 |
