# Supplementary material for: Collective movement of schooling fish reduces the costs of locomotion in turbulent conditions
Source: PLoS Biol. 2024 Jun 6;22(6):e3002501. doi: 10.1371/journal.pbio.3002501 (PMC11156351; doi:10.1371/journal.pbio.3002501)
Supplement: Attachment — Submitted filename: zhang-plosbio-2024Rev-v1.pdf [file pbio.3002501.s002.pdf]

REVIEW OF COLLECTIVE MOVEMENT OF SCHOOLING FISH REDUCES LOCOMOTOR COST IN TURBULENCE BY ZHANG ET AL.

GENERAL

The idea here is that swimming as part of a densely-packed school can lower locomotion costs when the environment is turbulent. A series of experiments were conducted to measure metabolic costs through O_2 consumption for solitary and 6-member schools. The authors show quite convincingly that swimming costs are substantially lower, per fish, in schools than alone, and that this difference is much higher when in a turbulent surrounding.

The manuscript is very well-written and presented, and will make a notable contribution to the literature. There are a number of comments below that might help improve the clarity and focus of the manuscript.

DETAILS

P4, l63: Yes, viscosity is 50 times that of air. Density is about 1000 times that of air. Is the underlying assumption that the forces to be modified are entirely viscous in nature? Perhaps it would be better here to express the dynamical similarity through the usual Reynolds number.

P4, l113: Correct English: "similar rise of values of all these parameters in control flow condition"

Fig. 1: L/H should be L/D. Why does the shelter envelope stand off from the leading edge of the school? Is the lead fish sheltered?

P4, l107: What is a controlled flow? Is it laminar? (the answer is provided at the end of the caption for Fig. 2, but it ought to be identified in the text too). Can we have some more conventional measures of turbulence for the turbulent and 'controlled' flow? In Fig. 2 the contrast flow is termed laminar. Turbulence is often characterized by u'/U where u' is the fluctuating component of a velocity component, or perhaps of the velocity magnitude. Since fluctuations of course scale with U, then it should be normalized with U also. The top two images in Fig. 2 are not useful. There needs to be a decision here about what quantity to show (the little velocity vectors are mostly invisible). Finally, but most importantly, the quantities need to be defined clearly. What is the coordinate system, what are the velocity components, is this a 2 dimensional system, what are the axes of Fig 2A,B? It might be helpful to express an \omega as \omega*D/U where D is a characteristic fish depth (as in Fig. 1). This now compares eddy turnover times to flow advection times. Vorticity *components* need to be clearly identified.

Fig. 3: Some concerns above have been addressed here, and the turbulence intensity (def?) is roughly constant with flow speed, as suspected. Would it make sense now to normalize L with D? Now we have components of velocity in u and v, but it should be made clear again, in the main text or caption, exactly what these are. (Presumably experiments will be in a three-dimensional environment.)

P5, l126: English: "Based on the energy cascade framework of turbulence eddy scale"

P5, l131: Style point, perhaps not universally accepted, but since science is supposed to be observer-independent, a sentence that begins like "We discovered that aerobic metabolic rate …" is more appropriately worded "The metabolic rate …". This kind of construction could be fixed in numerous places in the manuscript.

Fig. 4: A) Is it true that the cost of swimming at high speed in laminar flow is higher in a school than for an individual?

Fig. 4: Is it possible to express the ordinate as a dimensionless cost of transport? Something like the well-known P/WV where P is the metabolic power, and W and V are weight and speed.

P6, l169: Why was it necessary to employ 'high-speed videography' to measure school shapes. Do they vary in time; is it necessary to have the shapes resolved in time?

Fig. 5: The lack of coordinate system becomes painfully clear where 3 axes of a volume are all labelled 'distance'. This distance may also be normalized by the tank/channel dimensions. Some photographs of the fish school shapes in turbulent and laminar flows at the same speed would be a helpful precursor to Fig. 5. Then there needs to be an explanation of the source of the geometric shapes and colors plotted vs distance, distance, distance.

Fig. 5C: Seems to be mis-labelled. (C) is labelled laminar and control. Those two are the same thing and it should be laminar vs. turbulent. The confusion stems from the original use of the word control instead of laminar, which would be preferable and more informative.

P6, l173: The fish school assumes a prolate spheroid shape in contrast to a streamlined structure? Most people would say that the prolate spheroid is streamlined. Is there an aspect ratio that systematically changes?

P7, l201: This statement is imprecise: "Fish schools in turbulence expend up to 79% less energy than fish under control conditions (Fig. 4F)." Does this mean the difference between schools in laminar and turbulent conditions, or with an individual in turbulent conditions?

P7, l203: Here appears a statement about damping of turbulence by closely-packed swimmers. That seems different from mere sheltering. Is there any evidence? Though it may seem that tighter schools allow for unchanged kinematics, how do we know this is actually so?

P8, l215: What is the meaning of this phrase: "downshifting most of the locomotor performance curve"

P8, l233: Here appears the notion of damping again: "schooling dynamics effectively dampens the additional metabolic costs of moving in the turbulent flow" but now it is used in the context of reducing a cost.

P8,l237: What is this U_{crit}? It eventually is noted on p16, but not really explained. If U_{crit} is used in the main text then some minimal description of what it represents should be included.

P9, l244-248: Very general remarks on costs of behavior seem rather obvious and could be trimmed. Arguably, this entire paragraph is either a summary or composed of very general statements that add little value. By the end of it, we still have no information on what constitutes damping of turbulence or how this is quantified.

P10, l300: The idea of a fish school acting as a bandpass filter for hydrodynamic disturbances is interesting but very speculative.

P12, l346: How does a fish select a turbulent length scale?

P18, eq.(1): mathematical notation does not include the '*' symbol.

RECOMMENDATION

This paper will eventually be excellent. I hope the authors can consider some of the remarks above for a revised version.
