# Supplementary material for: Collective movement of schooling fish reduces the costs of locomotion in turbulent conditions
Source: PLoS Biol. 2024 Jun 6;22(6):e3002501. doi: 10.1371/journal.pbio.3002501 (PMC11156351; doi:10.1371/journal.pbio.3002501)
Supplement: Attachment — Submitted filename: Response to Reviewers report PLOS Biology.04.docx [file pbio.3002501.s003.docx]

**General response**

We would like to thank the editor and three reviewers for their positive responses to our manuscript. Animals frequently move in turbulent conditions, and we believe that the “turbulence sheltering hypothesis” tested in this paper is a novel idea for why animals might move in a collective formation. We are glad to see that reviewers view our study as providing compelling experiments and high-quality data to show that swimming costs in fish schools under turbulent conditions are lower than swimming alone. We also appreciate reviewers who think that our study will substantially advance our understanding of the benefits of collective movement and the field of locomotor biomechanics. Below, we respond to each comment, provide the requested analyses, improve the presentation as suggested, and offer increased justification for our interpretations. Reviewer #1 requested two additions to the manuscript by adding an additional panel to the original Figure 7 and a power spectrum analysis of those data. We have added these analyses as follows. Figure 7 has all four traces and the requested new spectral analysis which we agree is quite interesting and thank Reviewer #1 for this suggestion. The new Figure 8 just has the scaling analysis which was previously Figure 7A. We also added the kinematic analytic model suggested by Reviewer #1 in the Discussion. Reviewer #2 suggested a useful revision to Figure 1, and we did that. We also followed the suggestion to make the vector arrows clearer in panels a and b of Figure 2. In response to Reviewer #3, we explain our use of shear stress and the sum of shear stress as metrics of turbulence. Overall, we found the comments to be very constructive and helped to elevate the quality of the manuscript, and we very much appreciate the efforts of the editors and reviewers.

Below we provide each reviewer’s comment in black text and our response below in blue text.

Reviewer #1:

Dear authors,
I believe your paper represents a substantial advance in understanding the benefits of collective locomotion with a set of compelling experiments and high-quality data. I also don’t think that the presentation of these results matches the quality of the experiments, reducing the impact of your work and making your own interpretation of your results less clear than it should be. There are also some improvements to be made in the analysis of the kinematic data to better relate them to the energetics.

Response: We appreciate your input on improving the presentation of the results to match the quality of experiments and increase the potential impacts of our work. We attended to all analytical recommendations as detailed in the responses below. We believe the suggested kinematic analyses are very helpful for providing a deeper understanding of the energetics of animal locomotion and we plan to pursue this further in future studies.

Analysis:
First, some comments on tailbeat frequency and amplitude: As you’re aware, fluid forces increase with velocity^2. Thus, a simplified model suggests that, all else held equal, a 20% increase in tailbeat frequency would produce a 1.2^2 = 1.44 or 44% increase in force. Furthermore, because mechanical power is given by force * speed and speed increases linearly with frequency, the simple model of expected mechanical power (and muscle power) (and energy expenditure) for that 20% increase in tailbeat frequency is 1.2^2 * 1.2 = 1.2^3 = 1.73. Thus, cost ~ (frequency*amplitude)^3. Note that this model doesn’t account for speed from the fish’s forward motion but is at least a more physically realistic starting point for your attempt at comparing the kinematic and energetic changes around line 244.
Second, the head oscillations in Figure 7b are an intriguing qualitative look at how schooling affects swimming kinematics, but 1) the "school laminar" condition is missing and 2) the analysis could be made more quantitative by showing the power spectrum instead of just the time series. This should also be presented in the Results, not as an add-on in the Discussion.

Response:

Thanks for your thoughts on the simple but effective model of tailbeat frequency and amplitude. We agree with your reasoning and have added this to our discussion in the text around line 244 (line number in the original manuscript).

And thanks for your comment that we had not included the panel showing the “school laminar’ condition in Figure 7. We apologize for omitting this from the original manuscript. This was a mistake on our part, and the revised Figure 7 has added this panel. We also very much appreciate your great suggestion to do a power spectral analysis of these kinematic data. We did this and we have added a new Figure 7b in the revised manuscript to show the spectrum for the four different conditions. Thinking about the changes in head oscillation power spectra among the four tested conditions has prompted us to think about doing more analyses of this kind with kinematic data from our other schooling projects. We think that this approach could reveal some interesting and previously unexplored aspects of schooling dynamics in fishes.

Writing and presentation:
Please work to make your presentation of your results consistent, concise, and close to the actual results. For example, in this manuscript you refer to the same flow conditions as "controlled" and "laminar" on different occasions. Pick one! (I prefer laminar). Try and avoid discussion of topics not investigated in your work. For example, in lines 58-62 in only the second paragraph of the paper you digress toward discussing the effect of visual sensing on mitigating turbulence. You results have nothing to say about this and there is little other work to cite, so just go directly from "… alter body kinematics." To the new paragraph beginning with "This challenge…" Please review your text for similar digressions elsewhere.
Don’t rely on percentage comparisons, there are lots of ways to calculate them that give somewhat different numbers. PLoS Biology doesn’t restrict article length so there’s no reason not to provide the actual underlying measurement data.

Response:

These are all great suggestions.

We decided to pick “laminar” to describe the flow condition in the control group to minimize confusion by readers. We went back and forth on this ourselves and the only reason that we did not use laminar exclusively in the original manuscript is that we tested a wide range of speeds, and at high speeds in the flow tank the Reynolds number is high (30,000 or so), and the flow cannot be considered laminar in the classic definition. But the controlled flow condition, even at these higher tested speeds, certainly is much more “laminar” than the turbulent condition that we produced.

We removed the sentences discussing the visual sensing, and we have also now provided the actual underlying measurement data when we described the percentage comparisons.

Specific comments:
Line 27 - collective behavior is a vastly larger topic than locomotion, and many benefits are unrelated to animal movement. Substitute "benefits of collective movement" for "benefits of collective behaviours"

Response: We agree collective behaviour is a much larger topic than collective movement. Our edits clarify that we study collective movement as a part of understanding specifically the energetic benefits of collective behaviours. We change the opening statement of the abstract to “The ecological and evolutionary benefits of energy-saving in collective behaviours are rooted in the physical principles and physiological mechanisms underpinning animal locomotion.”

Line 34/35 - It is unclear what "reduced by 63%" means mathematically. English language is very imprecise when describing percentages, so you need to be thorough and provide a bit more context to help the reader decode your meaning. Consider instead "We discovered that, when swimming at high speeds in turbulent flows, fish in a school had only XX-YY% of the total energy expenditure of solitary fish." Alternatively, just provide the measurements, or at least do so along with your percentages.

Response: Thank you for suggesting the importance of clear communication. In this sentence, we stated that the percentage difference is a comparison to solitary fish. We believe this should be clear but to make the statement even more clear, we now provide the actual values of the measurements when describing the percentage difference. We inserted these measurement values throughout the manuscript.

Line 36 - see prior discussion about "kinematic effort". Also, this line is unrelated to the prior comparison of group effects. Consider introducing the effect of turbulence on solitary fish first, before describing how schooling helps reduce the effect.

Response: Thank you for the suggestions. We believe that presenting the key results first is a clear way of communicating the experiments. We then walk readers through presenting the comparisons with control groups that help us reach our conclusion. We hope reviewers can understand this rationale.

Line 37 - "However" suggests you’ll provide a follow-up to the prior sentence, but information about school 3D volume is unrelated to measures of individual tailbeat effort. Information about tailbeat effort in schooling fish would be appropriate following the "However", but volume is not.

Response: Thank you. Edited as suggested and we removed the word “However”. To clarify, we mentioned the kinematics of fish schools right after the schooling volume.

Line 40 – Didn’t you already discuss the effect of schooling versus solitary swimming on TEE back on line 34/35? Why say it again here with a different computation for the percentage result?

Response: We removed this sentence.

Summary: Your existing phrasing obscures your result. Consider "Swimming in a school substantially reduces the energetic cost of movement in turbulent water compared to swimming alone."

Response: We agree that the original sentence in the summary was confusing and have edited it to make it clearer. The revised sentence is ‘This substantial energy saving highlighted that schooling behaviour could mitigate turbulent disturbances by sheltering fish within schools from the eddies of sufficient kinetic energy that can disrupt locomotor gaits.’.

Line 61 - This line asserts that animals would have a hard time mitigating the energetic costs of turbulence without ever establishing (by citation or reasoning) that such costs exist. As mentioned before, the 2nd half of this first paragraph should be removed. What remains would flow logically to the second paragraph.

Response: Thank you for the suggestion. We removed the 2^nd^ half of this first paragraph.

Line 99: you write "whole animal", but your technique seems to measure "whole group" energy expenditure

Response: We understand the possible linguistic confusion, but in the fish energetics literature this technique is termed “whole animal respirometry”. We refined the technique to be able to measure one individual animal or a group of animals in a single experimental platform (this is explained in the Methods section). Although our schooling treatment group appears to measure the metabolic rate of a school of fish, the technique is still called “whole animal respirometry.”

Line 138: "… energy demands are at a premium." I think I understand your overall point, but this phrase doesn’t make sense to me - please rewrite.

Response: Thank you. We reworded this sentence as “Aerobic energy conservation enabled by schooling dynamics is more pronounced at higher speeds when the energy demands increased exponentially at higher speeds and when aerobic capacity is limited”.

Line 146: "1.8-fold faster" Why are you switching between reporting comparisons as percentages and as X-fold differences? Please be consistent in the way you report your results.

Response: We expressed all the comparisons to percentage differences for consistency.

Line 149: "62-79% lower" Again, your phrasing doesn’t make it immediately obvious what calculation you performed to get your results; please put the actual results in (like you did for EPOC results on line 145) or do a simpler computation, e.g. TEE and TCOT for fish in schools were XX-YY% of solitary fish. "% lower" makes it unclear what the denominator is in your computation.

Response: We do now present the actual values. Our original sentence stated that TEE and TCOT of fish schools were 62-79% lower than that of solitary individuals in turbulence. We are clear about the comparisons for the percentage difference.

Line 165 - again, badly reported percentages, better to have the actual numbers

Response: We now present the actual values.

Line 169 - is this result (smaller school size with speed) for turbulent or laminar flow or both?

Response: We now make it clear that fish schools in the turbulence have a smaller volume than the fish schools in the laminar flow at the match mean velocity. The 74% decline in 3-D school volume is in the turbulent condition. We also made this clear in the revision.

Line 177 - you use both f and f_tail to mean the same thing. Since the only amplitude discussed here is tailbeat amplitude, perhaps choose a more compact representation than "amp_tail" Greek letter theta would be good if your amplitude is an angle, but whether it is a linear or angular measure isn’t clear from the methods.

Response: We changed f to f_tail_ for consistency. Our tail beat amplitude is the linear distance, hence we will use Amp_tail_ as the abbreviation.

Line 202: is this supposed to read "less energy than [solitary] fish under control conditions" ?

Response: Thank you for spotting this! The sentence is now edited as “Fish schools in turbulence expend up to 79% less energy than solitary fish in turbulence.”

Line 202 - This statement is not supported by your results, how do you know that a closely packed school is an essential mechanism? It certainly happened, but you don’t have a comparison school that swam in turbulence without close packing to show the benefit of packing.

Response: This is an interesting point, but since we have replicates of fish schools and did not just study one school, we find that schools consistently show a smaller schooling volume as shown in Figure 5C. In other words, none of the schools in turbulent flow showed an increase or the same schooling volume when compared to laminar flow. This suggests that the reduced schooling volume is a key feature of fish’s response to turbulence. We do state that tighter schooling formation is “one of the essential mechanisms”, but not the only mechanism. By doing so, we acknowledge that future research can potentially discover more mechanisms that help fish schools to save energy in the turbulent environment.

Line 204 - Again, you don’t provide evidence that this is a result of closer packing

Response: We appreciate your raising this very interesting point. We addressed this point in the comments above and noted that we consistently (in the replicate schools) observed this closer packing. We think that one important feature of our study is that we did not just study one school, but that we conducted completely separate yet simultaneous energetic measurements on distinct replicate schools under both control and turbulent conditions. Thus, we feel that responses of the schools that occurred repeatedly for each group represent a robust finding that we should highlight.

Line 223 - this paragraph re-states the results and should be reduced or removed

Response: We condensed this paragraph to 35% of the original length.

Line 235 - another paragraph that restates results instead of discussing them

Response: We condensed the result summary section. We also condensed two paragraphs into the first paragraph of ‘Schooling dynamics dampens the effects of turbulence’.

Line 244 - as noted earlier, your effort = frequency*amplitude model is fluid-dynamically naïve and inadequate to make any comparison with your metabolic measurements

Response: Great point. As stated above, we improved the kinematic analyses following the methods you suggested and provided a reference. We have modified the manuscript as indicated above to discuss your suggested alternative model. But we note that the use of frequency*amplitude to represent “kinematic effort” in a general sense is actually used in the literature and has a long history in the study of fish kinematics (e.g. Webb 1973, J. Exp. Biol.). The use of frequency*amplitude provides an analysis that allows comparison to previous work. In addition, we now discuss the second kinematic model as suggested by Referee 1 (see the second paragraph in the section of ‘Schooling dynamics dampens the effects of turbulence’.

Line 289 - this discussion of oscillation amplitude is interesting and informative, but you should present these Results in the Results section

Response: Great suggestion. We moved the head oscillation to the Result section and added the analysis you suggested.

Line 289 - I’m not sure what you mean here "Individuals within a school swimming in turbulence have rhythmical head oscillations and more distinct peaks than those under turbulent conditions", please rephrase.

Response: Good point. We rewarded this sentence as “Individuals within a schooling swimming in turbulence have rhythmical head oscillations than those under turbulent conditions.” Thanks to this reviewer’s suggestion, we now have the power spectral analysis presented as a new Figure 7b, we have added some additional discussion of head oscillation frequency both in the Results section and in the Discussion sections.

Line 293 - if a tighter schooling volume is good and promotes all kinds of energy saving, why don’t schools in laminar flow also use the tighter volume? Wouldn’t they also like to save energy?

Response: Great point. School volume in laminar flow also declined as speed increased, but not as drastically as the decline showing up in turbulent flow. Our interpretation is that turbulence is a more challenging environment than the laminar flow all things being equal. Turbulent flow hence increases the likelihood of observing more specialized behaviours for energy saving where fish are under hydrodynamic stress. The intriguing question is then why don’t fish swim in tight formation all the time even when speed is low? Fish schools in low speeds and laminar conditions display a more random aggregation that can have stronger behavioural reasons than hydrodynamic reasons. Fish schools in turbulent flow and at high velocity become a more organized system. The ‘motivator’ demonstrated in the present study is energy saving.

Line 314 - What about CFD simulation? Isn’t the Lauder group working with Rajat Mittal on just such a project?

Response: Yes, indeed. The Lauder lab is working on CFD analyses of fish schools, and a postdoc in the lab is a CFD specialist trained in the “Rajat Mittal school of analysis”. We agree that an integration of biological data with CFD simulation is an interesting project, and this approach is a part of other ongoing projects. Current CFD simulation still cannot mimic the schooling dynamics and individual kinematics of real fish where fish constantly change position through time. Hence, without fully understanding the phenomenon and the mechanisms happening in the actual biological situation, directly diving into the CFD would run the danger of conducting a scientific investigation under too many less-understood assumptions. To convey the message more clearly, we edited this sentence as kinematic and energetic data provide the best ‘direct’ evidence for the turbulence sheltering hypothesis.

Line 335 - Could you be more clear on how migrating in a group protects against heat, hypoxia and storms? What’s your mechanism?

Response: We removed the sentence. Although we have some hypotheses, extending the discussion to speculate the mechanisms for each scenario would dilute the focus of the current study on turbulence.

Line 405 - "growth of microbial." Growth of microbial what?

Response: We changed this to “bacterial growth” which is clearer.

Line 412 & elsewhere: "Phontron" should be "Photron"

Response: Thank you, we changed “Phontron” to “Photron”.

Figure 4: it was not at all clear to me that the "model" lines refer to integrated aerobic and non-aerobic costs, please add this information to the figure itself and put it earlier in the caption

Response: Great point. We added, at the starting point of the paragraph, that model lines are the integrated aerobic and non-aerobic costs.

Figure 5: the figure legend specifies a comparison of "control" and "laminar", but aren’t those the same thing in your work?

Response: Thank you for spotting this possible confusion. We corrected the figure legend for the orange line to ‘school turbulence’.

Figure 6: please equalize the axis limits between left and right versions of the same measurement type

Response: Great point. We set the range of the Y-axis to be the same between the left and right panels.

Reviewer #2:
[IMPORTANT: see attachment for formatted version]

GENERAL

The idea here is that swimming as part of a densely-packed school can lower locomotion costs when the environment is turbulent. A series of experiments were conducted to measure metabolic costs through O2 consumption for solitary and 6-member schools. The authors show quite convincingly that swimming costs are substantially lower, per fish, in schools than alone, and that this difference is much higher when in a turbulent surrounding. The manuscript is very well-written and presented, and will make a notable contribution to the literature. There are a number of comments below that might help improve the clarity and focus of the manuscript.

Response: Thank you for regarding our manuscript as a piece of very well-written work that will contribute to the advancement of the study of collective behaviour. We agree that the comments below will greatly improve the manuscript, hence we attended to all comments and noted how we revised the manuscript accordingly.

DETAILS

P4, l63: Yes, viscosity is 50 times that of air. Density is about 1000 times that of air. Is the underlying assumption that the forces to be modified are entirely viscous in nature? Perhaps it would be better here to express the dynamical similarity through the usual Reynolds number.

Response: We agree that the Reynolds number (Re) is the best simple summary of this idea and we now include the range of Re (6.4•10^3^ – 1.8•10^5^) in the Methods to express the dynamical similarity. The point that we were trying to make is just that in the water there may be a premium for animals to find ways to save energy during locomotion since the resistive forces are so much higher than for air.

P4, l113: Correct English: “similar rise of values of all these parameters in control flow condition”

Response: Thank you and correct to “similar rise of all these parameters in laminar flow condition”.

Fig. 1: L/H should be L/D. Why does the shelter envelope stand off from the leading edge of the school? Is the lead fish sheltered?

Response: Very good point. We updated L/H to L/D. We trimmed the shelter envelope in Figure 1 to start in the mid-body, just behind the dorsal and anal fins of the lead fish. The leading fish may in fact be sheltered in the sense that a leading fish in front of other fish swimming behind can experience reduced locomotor costs (Thandickal and Lauder, 2023), but we do not know if this applies in this specific case of our hydrodynamic sheltering hypothesis, so we have modified the figure accordingly. Our current research shows that the dorsal and anal fins shed vortices and that the flow from these could also modify turbulence in the wake and so we have placed the new start of the sheltering envelope just behind these fins.

P4, l107: What is a controlled flow? Is it laminar? (the answer is provided at the end of the caption for Fig. 2, but it ought to be identified in the text too). Can we have some more conventional measures of turbulence for the turbulent and ‘controlled’ flow? In Fig. 2 the contrast flow is termed laminar. Turbulence is often characterized by u’/U where u’ is the fluctuating component of a velocity component, or perhaps of the velocity magnitude. Since fluctuations of course scale with U, then it should be normalized with U also. The top two images in Fig. 2 are not useful. There needs to be a decision here about what quantity to show (the little velocity vectors are mostly invisible). Finally, but most importantly, the quantities need to be defined clearly. What is the coordinate system, what are the velocity components, is this a 2 dimensional system, what are the axes of Fig 2A,B? It might be helpful to express an \omega as \omega*D/U where D is a characteristic fish depth (as in Fig. 1). This now compares eddy turnover times to flow advection times. Vorticity *components* need to be clearly identified.

Response: We have removed the text “controlled flow” and replaced it with “laminar flow” as we agree that this is confusing text. We have revised the top two images in Figure 2 to with larger yellow velocity vectors that are much more visible, and we do already provide the fluctuation velocity and normalized fluctuations (i.e. turbulence intensity) in Fig. 3C. In the Methods section we have added text to explain the coordinate system (yes, it is two-dimensional) and the caption to Figure 2 now explains this and the axes of panels A and B. We also clarify the components of the vorticity plotted in the caption to Figure 2.

Fig. 3: Some concerns above have been addressed here, and the turbulence intensity (def?) is roughly constant with flow speed, as suspected. Would it make sense now to normalize L with D? Now we have components of velocity in u and v, but it should be made clear again, in the main text or caption, exactly what these are. (Presumably experiments will be in a three-dimensional environment.)

Response: We provided the definition of turbulence intensity in the manuscript and in the Figure 3 legend. We agree that turbulent flow is three-dimensional, but we quantified this with 2D PIV. To clarify (as now revised in the figure caption), v is the horizontally perpendicular flow to the swimming direction of the fish, and u is the flow velocity in the free stream direction. We modified the Figure 3 caption to indicate that the ratio of L/D is about 1.

P5, l126: English: “Based on the energy cascade framework of turbulence eddy scale”

Response: Thank you and updated to “Based on the framework of turbulence scales and energy cascade”.

P5, l131: Style point, perhaps not universally accepted, but since science is supposed to be observer-independent, a sentence that begins like “We discovered that aerobic metabolic rate …” is more appropriately worded “The metabolic rate …”. This kind of construction could be fixed in numerous places in the manuscript.

Response: We agree that science is observer-independent. Using active voice is actually a conscious decision to make the sentence more interesting and easier to read. This writing style is recommended by leading scientific journals. We prefer to use the active voice where possible to engage the reader. We understand that not everyone will agree with this choice, however!

In this particular sentence, we are also conscious of the use of the verb ‘discovered’. This is because the observation stated in this sentence has not been published before, to the best of our knowledge. In this case, a passive voice would make the sentence harder to read. This being said, we went through the manuscript again and removed some not-so-unnecessary use of active voice. We hope that our revisions will still adhere to the principle that science is supposed to be observer-independent.

Fig. 4: A) Is it true that the cost of swimming at high speed in laminar flow is higher in a school than for an individual?

Response: The *aerobic performance* of the fish school swimming in laminar flow is higher than that of solitary individuals in laminar flow. However, the *total* energy expenditure of fish schools is much lower than that of the solitary individual (please see Fig. 2F & 2G).


Fig. 4: Is it possible to express the ordinate as a dimensionless cost of transport? Something like the well-known P/WV where P is the metabolic power, and W and V are weight and speed.

Response: We see the reviewer’s point, but we believe that our measurements convey the same meaning and that our current graph axis is more precise. We measured the metabolic rate used for locomotion but not the direct mechanical power generated by locomotor motion. The term metabolic power is hard to define at the organismic level. Biological systems at the organismic level use metabolic energy as a rate (or as a throughput), and our measurements use the rate of oxygen uptake to estimate the organismic metabolic rate. The word metabolic power at the organismic level is a bit like metabolic ‘rate’ ‘rate’. Although is perfectly fine to say the estimation of the metabolic power of muscle by force measurement, the measurements are based on mechanical power. Rest assured, our expression of the metabolic rate takes account of the weight, which is biomass, and speed. You can see both components are expressed in the unit of Kj km^-1^ kg^-1^.

P6, l169: Why was it necessary to employ ‘high-speed videography’ to measure school shapes. Do they vary in time; is it necessary to have the shapes resolved in time?

Response: The shapes of fish schools do indeed vary through time, and we decided to be cautious and obtain data at a higher rate than we probably needed for analyses. We could have taken still photographs at various times, but we believed that it was better to have a more continuous record of school shapes and fish relative positions. The speed of videography needed does depend on the research question. In this study, we are interested in the kinematics of individuals within the school as well as the school shapes. Hence, we use high-speed videography to record both. We then down-sample the video data specifically to understand the shape of the schools through time.

School shapes can be measured in a much lower frame rate of 5 frames per sec if the research question is only about what the overall schooling structure is. In fact, we are working on a project specifically to understand the 3-D shapes of schools through time in a high-resolution manner.

Fig. 5: The lack of coordinate system becomes painfully clear where 3 axes of a volume are all labelled ‘distance’. This distance may also be normalized by the tank/channel dimensions. Some photographs of the fish school shapes in turbulent and laminar flows at the same speed would be a helpful precursor to Fig. 5. Then there needs to be an explanation of the source of the geometric shapes and colors plotted vs distance, distance, distance.

Response: Figures 5A and B actually are based on a coordinate system. We clarify this point by updating the labelling as ‘Height, z’, ‘Width, y’, ‘Length, x’ in a unit of (mm). Different colours of convex hulls in one panel correspond to different sampling points of the same school at the same speed. We added the information to the figure caption.

Fig. 5C: Seems to be mis-labelled. (C) is labelled laminar and control. Those two are the same thing and it should be laminar vs. turbulent. The confusion stems from the original use of the word control instead of laminar, which would be preferable and more informative.

Response: Thank you! We updated the figure legends.

P6, l173: The fish school assumes a prolate spheroid shape in contrast to a streamlined structure? Most people would say that the prolate spheroid is streamlined. Is there an aspect ratio that systematically changes?

Response: We removed the statement about ‘prolate spheroid’ at the first paragraph of the kinematic section in Results. After careful consideration, we decided that it did not accurately describe what was observed. At this point, our data can only confidently show that fish schools are more densely packed. Thank you for pointing this out.

P7, l201: This statement is imprecise: “Fish schools in turbulence expend up to 79% less energy than fish under control conditions (Fig. 4F).” Does this mean the difference between schools in laminar and turbulent conditions, or with an individual in turbulent conditions?

Response: We revised the sentence to make it clear that the difference is the comparison to an individual in turbulent conditions.

P7, l203: Here appears a statement about damping of turbulence by closely-packed swimmers. That seems different from mere sheltering. Is there any evidence? Though it may seem that tighter schools allow for unchanged kinematics, how do we know this is actually so?

Response: ‘Dampen’ means reduced in this sentence. We showed that the metabolic costs are reduced and the extent of kinematics changes are also reduced when comparing the fish schools with the solitary individuals in the turbulence. To make the sentence more concise, we changed the verb from ‘dampen’ to ‘reduce’.

P8, l215: What is the meaning of this phrase: “downshifting most of the locomotor performance curve”

Response: To make the sentence more precise, we changed it to “downshifting the portion of the locomotor performance curve at the top 50% of the tested speed range”.

P8, l233: Here appears the notion of damping again: “schooling dynamics effectively dampens the additional metabolic costs of moving in the turbulent flow” but now it is used in the context of reducing a cost.

Response: ‘Dampens’ was used as a synonym for ‘reduces’. To avoid confusion, we changed this word to ‘reduces’.

P8,l237: What is this Ucrit? It eventually is noted on p16, but not really explained. If Ucrit is used in the main text then some minimal description of what it represents should be included.

Response: We understand that all readers may not be familiar with the Ucrit term (critical swimming speed), as it is specific to fish exercise studies, so thanks for requesting clarification. The idea of the Ucrit test is the same as a graded speed test (treadmill or stationary bike) on human exercise physiology. Ucrit is the maximum sustained swimming speed. It is a well-established concept that was first coined by Brett in 1964 and later refined by Farrell over the last three decades. In the first place we mentioned the critical swimming speed test (Ucrit), we added the definition of Ucrit (maximum sustained swimming speed) and provided a few more key citations.

P9, l244-248: Very general remarks on costs of behavior seem rather obvious and could be trimmed. Arguably, this entire paragraph is either a summary or composed of very general statements that add little value. By the end of it, we still have no information on what constitutes damping of turbulence or how this is quantified.

Response: We have condensed this text. We totally agree with the reviewer’s point about behaviours and metabolic costs. Although the costs of behaviours are not the same as the actual metabolic costs for the reviewer (who can be an expert in biomechanics), most biologists see that behavioural differences are always proportional and translate to the difference in metabolic costs. We want to highlight that this is an unfounded assumption.

To really understand what constitutes the reduction of turbulence, we will need to test the hypothesis that fish schools as hydrodynamic filters. This hypothesis is included in the discussion to offer some insights into the mechanisms. Future studies are much needed on this topic, considering fish schooling and turbulence are both ubiquitous in nature.

P10, l300: The idea of a fish school acting as a bandpass filter for hydrodynamic disturbances is interesting but very speculative.

Response: Thank you for acknowledging the bandpass filter hypothesis as being interesting and we agree that this is speculative. However, we would like to keep this text as it sets up the basis for future experiments to directly measure hydrodynamic filtering. We believe that a logical extension of the hydrodynamic sheltering hypothesis examined here is to explore the mechanism, and testing the hydrodynamic bandpass filtering hypothesis can explain one of the potential mechanisms.

P12, l346: How does a fish select a turbulent length scale?

Response: This is a very interesting question, and it is not known if fish do actually select a turbulent length scale in nature. In our experiments, we control the turbulent length scale to be challenging enough for the fish but not overly difficult that deter fish from swimming. In nature, we hypothesize that fish would show different responses to different turbulence length scales. If swimming through a region of flow with a manageable turbulent length scale means better access to food and mating opportunities, fish might try to go through the region of turbulent flow. Although these scenarios can be happening in nature on a daily basis, this avenue of research is still in its infancy. We will need many more studies to understand how the turbulent length scale can affect the ecology of fish swimming.

P18, eq.(1): mathematical notation does not include the ‘*’ symbol.

Response: We updated the ‘*’ symbol in the mathematical equation to the ‘•’ symbol throughout the manuscript.

RECOMMENDATION

This paper will eventually be excellent. I hope the authors can consider some of the remarks above for a revised version.

Response: Thank you!

Reviewer #3:

This is, to my mind, a really excellent paper. The results and conclusions are compelling; the findings and conclusions are important to the field of locomotion biomechanics, and the works is clearly presented and well supported. I have only minor comments on the manuscript.

Response: Thank you for seeing the significance of our study in the field of collective behaviour and locomotion biomechanics. We very much appreciate the positive review, and we have incorporated your suggested comments and edits as indicated below.

p4 - Turbulence results: Turbulence results are usually shown as a % of the average velocity, not as an absolute quantity - would this be possible?

I am not familiar with the characterization of the maxium and sum "shear strength" - can the authors provide some interpretation of this measure of the turbulence. More common is a measure of the shear vs rotational component of the flow, which can be assessed either from the "Swirl" or from the "Q" (See, for example Adrian et al, Expt Fluids 2000, and Jeong and Hussein, JFM 1995) - is there a connection between the "shear strength" and these more common measures of turbulence?

Response: We have added the definition and interpretation for the ‘shear strength’ measurements (maximum and sum) in the Methods section. We are using the approach advocated by Adrian, R. J., Christensen, K. T. and Liu, Z.-C. (2000). Analysis and interpretation of instantaneous turbulent velocity fields. Experiments in Fluids 29, 275-290, and using the result as calculated by the Lavision DaVis 8.3.1 software. The % of the average velocity which is equivalent to the turbulence intensity is actually shown in Figure 3, panel C. Note that this quantity does not change much as it is coupled to the mean flow (and is thus not a particularly useful metric). Maximum shear strength is just the maximum value of shear strength calculated from the 2D velocity fields, and since the shear can be positive and negative we also report the sum of the squared values in panel F to provide another metric of how fluid shear changes with speed and in the comparison between the control and turbulent conditions.

p6 L174 - the phrase "controlled flow" doesnt seem quite right - its not controlled. I assume it means the control case, but even that seems cumbersome - would "quiescent flow", "clean flow", low-noise flow", "laminar flow" or something like that be better?

Response: Thank you, and indeed this is a bit confusing. We updated the text for controlled flow to laminar flow.

p8 L 237. The authors use the "kinematic effort" as a proxy to assess swimming effort - why? can they provide some background or previous result that demonstrates that power might be related to kinematic effort?

Response: The use of "kinematic effort" estimated tail beat amplitude times frequency has been used previously in the fish kinematic literature and has a classic history going back to the early fish kinematic studies in the 1970s (e.g. Webb 1973, J. Exp. Biol.) and we thought that it provided a simple metric that is a proxy for energy use from a kinematic point of view. To provide a more comprehensive view of the kinematic assessment for locomotion efforts, we added a further discussion point about another kinematic model to estimate the effort of locomotion.

p20 L 602 "Photron", not "Phontron"

Response: We corrected “Phontron” to “Photron” throughout the manuscript.


These are small objections. All in all the paper is remarkable, and I am happy to recommend publication

Response: Thank you!
